# *FMR1* expression in human granulosa cells increases with exon 1 CGG repeat length depending on ovarian reserve

**DOI:** 10.1186/s12958-018-0383-5

**Published:** 2018-07-07

**Authors:** Julia Rehnitz, Diego D. Alcoba, Ilma S. Brum, Jens E. Dietrich, Berthe Youness, Katrin Hinderhofer, Birgitta Messmer, Alexander Freis, Thomas Strowitzki, Ariane Germeyer

**Affiliations:** 1 Reproduction Genetics Unit, Department of Gynecological Endocrinology and Fertility Disorders, University Women’s Hospital, Heidelberg, Germany; 2 Department of Physiology, Institute of Health Sciences, Federal University of Rio Grande do Sul, Porto Alegre, Brazil; 3 Department of Gynecological Endocrinology and Fertility Disorders, University Women’s Hospital, Heidelberg, Germany; 4 Laboratory of Molecular Genetics, Institute of Human Genetics, Heidelberg University, Heidelberg, Germany

**Keywords:** *FMR1* expression, Granulosa cells, *FMR1* CGG repeat length, *FMR1* genotype

## Abstract

**Background:**

*Fragile-X-Mental-Retardation-1-* (*FMR1*)*-gene* is supposed to be a key gene for ovarian reserve and folliculogenesis. It contains in its 5’-UTR a triplet-base-repeat (CGG), that varies between 26 and 34 in general population. CGG-repeat-lengths with 55–200 repeats (pre-mutation = PM) show instable heredity with a tendency to increase and are associated with premature-ovarian-insufficiency or failure (POI/POF) in about 20%. *FMR1-*mRNA*-*expression in leucocytes and granulosa cells (GCs) increases with CGG-repeat-length in PM-carriers, but variable *FMR1-*expression profiles were also described in women with POI without PM-*FMR1* repeat-length. Additionally, associations between low numbers of retrieved oocytes and elevated *FMR1*-expression levels have been shown in GCs of females with mid-range PM-CGG-repeats without POI. Effects of *FMR1-*repeat-lengths-deviations (*n* < 26 or *n* > 34) below the PM range (*n* < 55) on ovarian reserve and response to ovarian stimulation remain controversial.

**Methods:**

We enrolled 229 women undergoing controlled ovarian hyperstimulation for IVF/ICSI-treatment and devided them in three ovarian-response-subgroups: Poor responder (POR) after Bologna Criteria, polycystic ovary syndrome (PCO) after Rotterdam Criteria, or normal responder (NOR, control group). Subjects were subdivided into six genotypes according to their be-allelic CGG-repeat length. *FMR1*-CGG-repeat-length was determined using ALF-express-DNA-sequencer or ABI 3100/3130 × 1-sequencer. mRNA was extracted from GCs after follicular aspiration and quantitative *FMR1*-expression was determined using specific TaqMan-Assay and applying the ΔΔCT method. Kruskall-Wallis-Test or ANOVA were used for simple comparison between ovarian reserve (NOR, POR or PCO) and CGG-subgroups or cohort demographic data. All statistical analysis were performed with SPSS and statistical significance was set at *p* ≤ 0.05.

**Results:**

A statistically significant increase in *FMR1-*mRNA-expression-levels was detected in GCs of PORs with heterozygous normal/low-CGG-repeat-length compared with other genotypes (*p* = 0.044).

**Conclusion:**

Female ovarian response may be negatively affected by low CGG-alleles during stimulation. In addition, due to a low-allele-effect, folliculogenesis may be impaired already prior to stimulation leading to diminished ovarian reserve and poor ovarian response. A better understanding of *FMR1* expression-regulation in GCs may help to elucidate pathomechanisms of folliculogenesis disorders and to develop risk-adjusted treatments for IVF/ICSI-therapy. Herewith *FMR1-*genotyping potentially provides a better estimatation of treatment outcome and allows the optimal adaptation of stimulation protocols in future.

## Background

A sufficient ovarian reserve is crucial for female fertility and, consequently, a successful pregnancy. Diminished ovarian reserve can considerably affect the success rates of assisted reproductive techniques (ARTs) [1]. The response to controlled ovarian hyperstimulation, in addition to oocyte quality, reflects female reproductive potential. Therefore, understanding the mechanisms underlying ovarian response patterns are necessary for improving ART approaches.

*FMR1* (fragile X mental retardation 1) gene is one of the major genes of interest in this field, as it is associated with premature ovarian insufficiency (POI) and its endpoint premature ovarian failure (POF; OMIM accession number: 615723) and, as its protein FMRP is mainly localized in granulosa cells within the ovary [2, 3]. The *FMR1* gene contains a CGG repeat of variable size (generally, approximately 30 repeats long) in its 5′-untranslated region (UTR) of exon 1 [4]. If the repeat length extends over 200 (full mutation: FM), individuals can develop the fragile X syndrome (OMIM accession number: 300624), which is linked with a mental retardation caused by *FMR1* gene silencing and loss of fragile X mental retardation 1 protein (FMRP) [5]*.* While FM-carriers do not show an increased risk for the development of POI/POF, premutation (PM) carriers frequently (~ 20%) suffer from this disorder [2], known as fragile X-associated POI (FXPOI) as well. They have > 54 and < 200 CGG repeats in their *FMR1* gene and demonstrate a repeat length instability with a tendency of increasing repeat lengths from one generation to the next.

In leukocytes and lymphoblastoid cells of male and female *FMR1* PM carriers, *FMR1* mRNA was shown to be overexpressed, while its protein level was decreased [6, 7]. This inverse correlation can be explained by the presence of a regulatory feedback mechanism, where high levels of *FMR1* mRNA may be toxic and lead to the development of pathologies in PM carriers [8]. For the PM-associated neurological disorder FXTAS (fragile X-associated tremor/ataxia syndrome) symptoms are explained by the formation of intranuclear inclusions by the extended CGG triplet block, that result from aberrant protein binding to specific hairpin structures within the nucleus. Sequestration of the *FMR1*-PM mRNA thereby leads to elevated transcription rates [9, 10]. Although similar results and mechanisms are expected in women with POI as well, extensive studies on women and on female germline cells have not been conducted. Previously, we detected alterations in *FMR1* expression levels in leukocytes of women with POI, which were shown to be independent of the PM status [3]. Chen et al. demonstrated that CGG repeat length, regardless of the PM status, in human neuronal and kidney cells may act as positive or negative modulators of *FMR1* translation [11]. Additionally, several studies demonstrated that CGG repeats below 26 or above 34 may affect ovarian reserve and fertility as well [12–16]. According to the repeat lengths at both alleles (low < 26 repeats; normal 26–34 repeats; high 35–55 repeats) women can be divided into six different *FMR1*-CGG-genotypes: high/high, high/low, normal/high, normal/normal, normal/low, and low/low [17]. A pathological effect of CGG repeats outside the range of 26–34 repeats remains controversial, since it was not observed in studies with different experimental settings [18–22].

*FMR1* expression levels in leukocytes and other cell types may not be equivalent to the levels in germline cells. During folliculogenesis, oocytes are surrounded by granulosa cells (GCs), forming a functional entity. These cells are necessary for the proper development of oocytes before ovulation. In the human ovaries, GCs represent the main source of FMRP [3], which led us to analyze *FMR1* mRNA expression directly in human GCs, thereby avoiding potential bias originating from the usage of animal models or different human cell types or lines. This study aimed to evaluate the effects of different *FMR1* genotypes, according to the allele specific CGG repeat length, on the expression of this gene in GCs in an ovarian response-dependent manner.

Elizur et al. demonstrated a significant non-linear association between CGG repeat length and *FMR1* expression levels in GCs of female PM carriers, with the highest *FMR1* expression level in women with mid-range CGG repeat length (80–120 triplets), which was shown to be associated with a low number of oocytes retrieved during in vitro fertilization (IVF) [23]. Mid-range PM carrier status is also supposed to demonstrate the highest risk for developing POI/POF in women [24].

To the best of our knowledge, association studies analyzing *FMR1* mRNA expression profiles in GCs with aberrant *FMR1* CGG repeat numbers below the PM threshold have not been performed to date. Therefore, we evaluated the effects of the six *FMR1* genotypes (i.e. low, normal and high repeat numbers for each allele) on the *FMR1* mRNA expression profile in GCs, using GCs obtained from women with different ovarian response patterns: women with poor ovarian response (POR), women with normal ovarian function (NOR), and women with polycystic ovary syndrome (PCOS).

## Methods

We aimed to evaluate if *FMR1-*CGG-repeat-lengths aberrations from normal range (n: 26–34) influence the mRNA-expression in GCs of women in an ovarian response depending manner.

### Study population

A total of 229 women that underwent controlled ovarian hyperstimulation for either IVF or IVF with intracytoplasmic sperm injection (ICSI) treatment at the Department of Gynecological Endocrinology and Reproductive Medicine, University Women’s Hospital, Heidelberg, from February 2013 to August 2016 were prospectively recruited for our study. We collected GCs and blood samples from all patients. Additionally, their medical records and questionnaires were assessed in order to obtain demographic information (age at presentation and body mass index [BMI]), baseline hormone levels (serum follicle stimulating hormone [FSH], luteinizing hormone [LH], estradiol [E_2_], and anti-Müllerian hormone [AMH]), and reproductive parameters (antral follicle count [AFC], total number of oocytes recovered, and mature [MII] oocytes retrieved). Patients were divided into three response groups. According to the Bologna Criteria [25], patients were included into the POR group (*n* = 70); in case of clinically documented PCOS, according to the Rotterdam Criteria [26], patients were included in the PCO group (*n* = 8). Those who did not fulfill criteria for POR or PCO groups were included in the NOR group, which served as a control (*n* = 151).

### Ethical approval

All patients signed an informed consent form and completed a clinical questionnaire. This study was approved by the local ethical committee of the University of Heidelberg, Germany (number S-602/2013), and conducted according to the principles expressed in the Declaration of Helsinki.

### CGG repeat length analysis

DNA samples were prepared as described previously [27] from 10 ml of blood samples with EDTA. To analyze CGG repeat length in the 5′-UTR of *FMR1* exon 1, polymerase chain reaction (PCR) analysis and subsequent analysis of this region with the ALFexpress DNA sequencer (Amersham 1050; Pharmacia Biotech, Freiburg, Germany) or ABI 3100/3130xl sequencer (Life Technologies/Applied Biosystems, Foster City, CA, USA) were performed. PCR mixture (total volume, 30 ml) contained 0.25 μM of each primer (for forward and reverse primer sequences see Fu et al., 1991), 0.2 mM of dATP, dCTP, and dTTP each, 50 μM dGTP, 150 μM deaza-dGTP, 0.12 U KAPA Hot Start Taq polymerase, 1× PCR buffer, 1.5 mM MgCl_2_, 1× Enhancer (Qiagen GmbH, Hilden, Germany), and 50 ng of genomic DNA. PCR conditions were as follows: 3 min at 94 °C for the first denaturation step; 35 cycles of amplification with a time-temperature profile of 15 s at 94 °C, 15 s at 66 °C, 15 s at 72 °C; and the additional incubation for 8 min at 72 °C in the last cycle. The forward primer was labeled with the fluorescent Cy5 or FAM dye (Eurofins Genomics, Ebersberg, Germany). For the analysis using ALFexpress sequencer, a 5 μl aliquot of PCR mix was mixed with 5 μl of 6× loading solution (5 mg/ml Blue Dextran (Carl Roth GmbH + Co. KG, Karlsruhe, Germany) in formamide (Merck KGaA, Darmstadt, Germany)) and 1 μl of 250-bp internal marker. All samples following the denaturation at 95 °C for 5 min were analyzed on 6% denaturing polyacrylamide gel with 7 M urea. A 70–397 nucleotide size marker labeled with Cy5 dye was used for the determination of CGG repeat numbers. Allele sizes and peak areas of fluorescent products were analyzed with the Fragment Manager software (Pharmacia Biotech, Freiburg, Germany). To analyze the samples on the ABI 3100/3130xl sequencer, 1 μl of PCR product was mixed with 10.5 μl of Hi-Di-formamide and 0.5 μl of GeneScan ROX standard (Applied Biosystems, Foster City, CA, USA) and loaded. The obtained data were analyzed with the GeneMapper software (Applied Biosystems, Foster City, CA, USA). When the presence of PM was suspected, Southern blot was performed using a-^32^P-dCTP radioactively-labeled p2 probe containing *FMR1* exon 1 with CGG repeat as described previously [28].

### Non-PM allele length classification

A CGG repeat length of 26–34 was considered a normal repeat length range according to prior studies [4, 14–16, 20]. We classified the patients according to the repeat lengths at both alleles (low < 26 repeats; normal 26–34 repeats; high 35–55 repeats) into six different genotypes: high/high, high/low, normal/high, normal/normal, normal/low, and low/low, as previously described [17]. PM carriers were not included in the study.

### Ovarian stimulation

Ovarian stimulation was performed using either the long protocol of gonadotropin-releasing hormone (GnRH) agonist administration (long GnRH agonist protocol) or GnRH antagonist protocol. The appropriate protocol was selected by the physicians in charge. In the long GnRH agonist protocol an initial down-regulation using a GnRH-agonist at day 20+ 1 of the menstrual cycle was used. On day two of following cycle, gonadotropins (mainly recFSH or HMG) were injected daily to induce proper follicle maturation. When follicles reached 18 mm diameter, ovulation was induced by HCG injection and the oocytes were retrieved via ultrasound-guided follicular puncture after 36 h in 14 ml round bottom tubes containing PBS (1× Phosphate-buffered saline) and heparin (250 μl Heparin / 500 ml PBS). In the GnRH-antagonist protocol, gonadotropins (mainly recFSH or HMG) were injected daily to induce proper follicle maturation beginning at day 2 of menstrual cycle. When the leading follicle reached 14 mm average diameter, a GnRH-antagonist was used to prevent preterm spontaneous ovulation. At stage of 18 mm diameter follicle size, oocytes were retrieved after ovulation induction as described above. The cumulative dose of gonadotropins was determined based on the patients’ response and the decisions made by the physicians in charge.

### Retrieval of GCs

GCs were retrieved from the follicular fluid after the transvaginal ultrasound-guided follicle puncture for IVF treatment that was performed with an ovum aspiration needle (Premium Fas Single Lumen, #4551 NS-AS1; Gynétics Medical Products N.V., Lommel, Belgium) connected to a vacuum pump (Cook Medical, K-MAR-5200, Bloomington, IN, USA). The aspirated follicular fluid was collected in 14 mL round-bottom tubes (Falcon, 352,001, NY, USA), and kept at 37 °C in a test-tube heater (Cook Medical, K-FTH-1012, Bloomington, IN, USA) or in a Thermo-Cell-Transporter (Labotect, Thermo-Cell-Transporter 3018, Rosdorf, Germany). Follicular fluid was transferred to a 100-mm cell culture dish (Thermo Fisher Scientific, Nunc, Waltham, MA, USA) on a table heated to 37 °C (Workstation L126 Dual, K-Systems, Birkerød, Denmark). GCs were identified morphologically as epithelial cell aggregates within the follicular fluid using a Nikon SMZ1500 zoom-stereomicroscope (Nikon Instruments Europe B.V., Amsterdam, Netherlands). In most cases granulosa cells (GCs) were picked up directly from the follicular fluid without additional washing. A brief washing step in either Multipurpose Handling Medium (MHM) or Sydney IVF Fertilization medium (Cook, K-SIFM-20, Bloomington, IN, USA) was considered necessary, if the follicular fluid was bloody. In some cases, i.e. bloody follicular fluid and a lot of Cumulus-Oocyte-Complexes (COCs), the MHM used for keeping COCs during the search also became bloody. In those cases GCs were briefly washed in Sydney IVF Fertilization medium that was also used to wash COCs before in vitro culture. We did not check for pH variations in Sydney IVF Fertilization medium after washing GCs and therefore cannot exclude a possible impact. However, only equilibrated Sydney IVF Fertilization medium was used for washing of COCs. The Sydney IVF Fertilization medium was then used immediately to wash GCs. Thus, the time during which Sydney IVF Fertilization medium was used outside the CO^2^-incubator was kept to a minimum.

Mural GCs were aspirated in a 2.5 μl volume with a sterile tip (ep Dualfilter T.I.P.S. 10 μl S, Eppendorf, Wesseling-Berzdorf, Germany), transferred to 1.5-ml tubes (Sarstedt, Nümbrecht, Germany) pre-filled with 12–13 μl of RNAlater stabilization solution (Ambion, AM7020, Life Technologies, Carlsbad, CA, USA), and stored at 4 °C.

### RNA extraction

GCs in the stabilizing solution were centrifuged at 5000×*g* for 5 min, and the supernatants were removed. mRNA was directly isolated from the GCs using TRIzol (Life Technologies, Carlsbad, CA, USA) according to the manufacturer’s instructions [29, 30] with PEQGOLD PHASETRAP A 1.5 ml tubes (VWR International GmbH, Darmstadt, Germany). mRNA was dissolved in RNAse-free water and the concentration and purity were detected using NanoDrop 2000c UV-spectrometer (NanoDrop Products, Wilmington, DE, USA). cDNA samples were synthesized after oligo-dT priming with SuperScript III First-Strand Synthesis System (Invitrogen by Life Technologies, Carlsbad, CA, USA) and the M-MLV Reverse Transcriptase, RNase H Minus, Point Mutant (Promega, Madison, WI, USA).

### Gene expression analysis

TaqMan predesigned gene expression assays for *FMR1* (Hs00924544_m1) and two housekeeping genes, *HPRT* and *TBP* (Hs99999909_m1; Hs00427620_m1, respectively), as well as the TaqMan universal PCR master mix were purchased from Applied Biosystems (Life Technologies, Carlsbad, CA, USA) and the experiments were performed according to the manufacturer’s instructions. The samples were analyzed in triplicates, and standard PCR conditions were used, with Fast Forward 7500 real-time PCR-system (Applied Biosystems, Life Technologies, Carlsbad, CA, USA). Relative gene expression was analyzed using ΔΔCt method [31]. cDNA obtained from the COV 434 granulosa cells [32] was used as a calibrator in each run.

### Statistical analysis

Data distribution was first determined by Shapiro-Wilk-Test. For simple comparison between ovarian reserve- (NOR, POR, or PCO) or CGG-subgroups, and the analysis of cohort demographic data, Kruskall-Wallis test or analysis of variance (ANOVA) were used. When statistically significant differences were obtained between groups, a post hoc test [Tukey’s honestly significant difference (HSD) or Dunn’s tests] was performed to identify which of the analyzed subgroups differed. In order to adjust the significance level, the Bonferroni correction was applied. Additionally, the χ2-test, supplemented by the adjusted residuals, was used for between- group comparisons (clinical ovarian reserve classification and genotype). Results are presented as mean ± standard deviation (SD), or median and interquartile range (percentile 25-percentile 75; respectively 1^rst^-3rd quartile). For *n* < 3, the data are presented as median and minimum and maximum value (minimum–maximum). Statistical analyses were performed with SPSS (Statistical Package for the Social Sciences V. 22.0; IBM Corporation, NY, USA), and statistical significance was set at *p* ≤ 0.05.

## Results

### General study population

#### Cohort demographics

Of 229 patients participating in our study, 151 were classified as NORs, 70 belonged to the POR group, while eight patients were included in the PCO group (Table 1). No differences in BMI and estradiol (E_2_) levels, diagnosed at early follicular phase, were determined between the ovarian response groups. The age of patients of the 3 response groups differed whereby PCO patients were the youngest (Table 1).

**Table 1:** Cohort demographics

Demographic	NOR		POR		PCO		*p value*
	n	Median (P25-P75)	n	Median (P25-P75)	n	Median (P25-P75)	
Age*	151	35.1 ± 4.2^A^	70	37.4 ± 4.6^B^	8	31.3 ± 3.8^C^	0.001
BMI	149	23.6 (20.5–27.3)	65	22.3 (20.7–25.4)	8	26.3 (20.9–32.1)	0.352
AFC	84	6 (4.5–9)^A^	35	2.5 (1.5–3.5)^B^	6	16.25 (12.1–20)^C^	< 0.001
FSH (U/L)	137	7.2 (6–8.8)^A^	59	8.6 (6.1–11.1)^B^	7	6.1 (5.8–7.2)^AB^	0.013
LH (U/L)	139	5.3 (3.7–6.8)^A^	62	5.5 (3.5–6.5)^A^	8	12.9 (9.6–17.5)^B^	< 0.001
Estradiol	133	43 (34.5–58.2)	59	49.2 (31.1–76.0)	8	41.7 (31.1–46.4)	0.810
AMH	142	2.47 (1.4–3.8)^A^	69	0.88 (0.5–1.1)^B^	8	9.37 (4.5–14.6)^C^	< 0.001
Total oocytes	149	9 (6–13)^A^	64	3 (2–5.7)^B^	8	22 (4.2–36)^A^	< 0.001
MII oocytes	117	7 (5–11)^A^	43	3 (2–5)^B^	6	16 (8–27.2)^A^	< 0.001
*FMR1*	138	0.75 (0.5–1.2)	57	0.8 (0.5–1.2)	8	0.9 (0.46–1.43)	0.947

*BMI* body mass index, *AFC* antral follicle count, *FSH* follicle stimulating hormone, *LH* luteinizing hormone, *AMH* anti-Müllerian-hormone, *MII* oocytes, mature oocytes, *FMR1* fragile X mental retardation 1 gene relative gene expression in granulosa cells of the patients normalized by two house-keeping genes and a granulosa cell calibrator (see MM-part for details)

All other values represent median values, with 1st and 3rd quartile parenthesized

Different letters in one row signify statistical difference

*p* values represent significance levels between normal responders (NOR), poor responders (POR), and polycystic ovarian syndrome group (PCO)

*Mean ± standard deviation

As expected, FSH (*p* = 0.013), LH, AMH, AFC, the number of total oocytes retrieved, and the number of MII oocytes differed significantly (*p* < 0.001 for all; Table 1) between these three groups, which demonstrated the appropriate patient selection.

In the three groups treatment distribution between IVF, ICSI and IVF/ICSI-splitting was comparable between NOR and PCO with 69.3%, respectively 62.5% for ICSI-treatments and with 26.7%, respectively 25% for IVF-treatments. However, in the POR group the percentage of IVF was higher (45.2%) and no splitting was performed in our population.

#### FMR1 mRNA expression

Relative mRNA *FMR1* expression in GCs did not differ between the three response groups (*p* = n.s.; Table 1).

### *FMR1* genotype groups

#### Cohort demographics

To evaluate the potential effects of CGG repeat number aberrations on ovarian response and/or mRNA *FMR1* expression in human GCs of IVF/ICSI patients, we divided the patients according to their allele CGG repeat lengths into six genotype groups: high/high, high/low, normal/high, normal/normal, normal/low, and low/low groups.

As age distribution in all genotype groups was similar, we did not perform any further age-dependent analyses. FSH, LH, AMH, AFC, the number of total oocytes retrieved, and the number of MII oocytes did not differ between different genotype groups (*p* = n.s. for all; Table 2).

**Table 2:** Cohort demographic analysis of groups formed according to *FMR1* genotypes

	All patients												
Demographics	high/high		high/low		high/normal		normal/normal		normal/low		low/low		*p* value
	n	(P25-P75)	n	(P25-P75)	n	(P25-P75)	n	(P25-P75)	n	(P25-P75)	n	(P25-P75)	
Age^a^	2	34 ± 5.6	3	28.6 ± 1.1	25	35.4 ± 4.4	123	35.7 ± 4.5	65	35.9 ± 4.7	8	36.3 ± 3.2	0.163
AFC	2	6 (6–6)	0	–	18	3 (1.8–7.1)	62	5.75 (3–9.12)	38	5 (3–6.1)	3	5.5 (3.5–10)	0.239
FSH	2	9.5 (6.6–12.4)	2	7.3 (6.9–7.7)	23	8.3 (6.1–10.9)	110	7.1 (5.7–8.7)	56	7.8 (6.2–9.3)	7	7.9 (5.2–10.3)	0.323
LH	2	9.2 (6.7–11.7)	3	4.8 (4.7–6.1)	24	5.7 (3.7–10.1)	113	5.4 (3.5–7.1)	56	5.3 (3.7–6.4)	8	4.9 (3.6–6)	0.338
Estradiol	2	55.8 (51.5–68.2)	3	47.3 (31.2–63.1)	23	45.6 (38.2–76)	108	40.6 (32.4–58.2)	54	43.6 (34.3–57.9)	7	55.7 (41.5–76)	0.372
AMH	2	1.24 (0.94–1.55)	2	2.49 (2.48–2.5)	25	0.88 (0.55–3.35)	120	1.86 (1.06–3.68)	59	1.87 (1.14–3.16)	8	1.37 (1.03–1.64)	0.426
Total oocyte number	2	2.5 (2–3)	3	9 (7–14)	23	6 (2–14)	120	7 (4–12)	63	7 (5–12)	8	4.5 (3.2–6)	0.210
MII oocyte number	2	2 (2–2)	2	6.5 (6–7)	16	6 (2–15)	92	6 (3–9)	49	6 (4.5–9)	5	5 (3–8.5)	0.739

*BMI* body mass index, *AFC* antral follicle count, *FSH* follicle stimulating hormone, *LH* luteinizing hormone, *AMH* anti-Müllerian-hormone, *MII* oocytes mature oocytes.

^a^Age, mean ± standard deviation is presented

All other values are presented as median and 1st and 3rd quartile parenthesized

*p* values represent significance levels between the six evaluated *FMR1* genotypes

#### Ovarian response

The correlation analysis of the CGG genotypes and three ovarian response groups showed no significant differences in genotype distribution (*p* = n.s.; Table 3), demonstrating that different genotypes in general are not associated with the ovarian response in our study population.

**Table 3:** *FMR1* genotype distribution in patients with different ovarian response patterns

Frequencies	NOR - n (%)	POR - n (%)	PCO - n (%)
high/high	1 (0.7)	1 (1.4)	0 (0.0)
high/low	3 (2.0)	0 (0.0)	0 (0.0)
high/normal	13 (8.7)	10 (14.5)	2 (25.0)
normal/normal	79 (53.0)	38 (55.1)	6 (75.0)
normal/low	47 (31.5)	18 (26.1)	0 (0.0)
low/low	6 (4.0)	2 (2.9)	0 (0.0)

*NOR* normal responders, *POR* poor responders, *PCO* polycystic ovary syndrome patients

Ovarian response distribution among the six *FMR1* genotypes (*p* = 0.54)

#### FMR1 mRNA expression

Different genotypes in general were not shown to be related with *FMR1* expression levels (*p* = n.s.; Table 4). However, analysis of *FMR1* expression related to ovarian response showed that the normal/low genotype in PORs is significantly associated with an increase in *FMR1* expression in GCs (*p* = 0.044; Table 4).

**Table 4:** *FMR1* mRNA gene expression levels in different *FMR1* genotype and ovarian response groups (age-independent)

*FMR1* Expression	high/high		high/low		high/normal		normal/normal		normal/low		low/low		*p* value
	n	(P25-P75)	n	(P25-P75)	n	(P25-P75)	n	(P25-P75)	n	(P25-P75)	n	(P25-P75)	
All patients	1^a^	0.309	3	0.32 (0.26–1.54)	21	0.67 (0.51–1.21)	109	0.73 (0.47–1.25)	59	0.84 (0.55–1.27)	8	0.68 (0.19–1.69)	0.475
NOR	1^a^	0.309	3	0.32 (0.26–1.54)	10	0.95 (0.53–1.47)	71	0.71 (0.48–1.25)	45	0.77 (0.52–1.09)	6	0.68 (0.16–1.9)	0.630
POR	0	–	0	–	9	0.56 (0.47–0.69)	32	0.78 (0.43–1.2)	14	1.1 (0.95–1.5)	2	0.77 (0.2–1.3)	0.044
PCO	0	–	0	–	2	1.05 (0.92–1.19)	6	0.83 (0.35–1.53)	0	–	0	–	0.505

Distribution of *FMR1*-expression in GCs among the six genotypes related to different ovarian response groups. Normal/low genotypes demonstrated elevated *FMR1* expression in case of poor response (*p* = 0.044)

^a^Descriptive analysis only, due to the number of patients

*p* values represent significance-levels between the six genotypes and are calculated as described in the Material and Method part

**Fig. 1:**
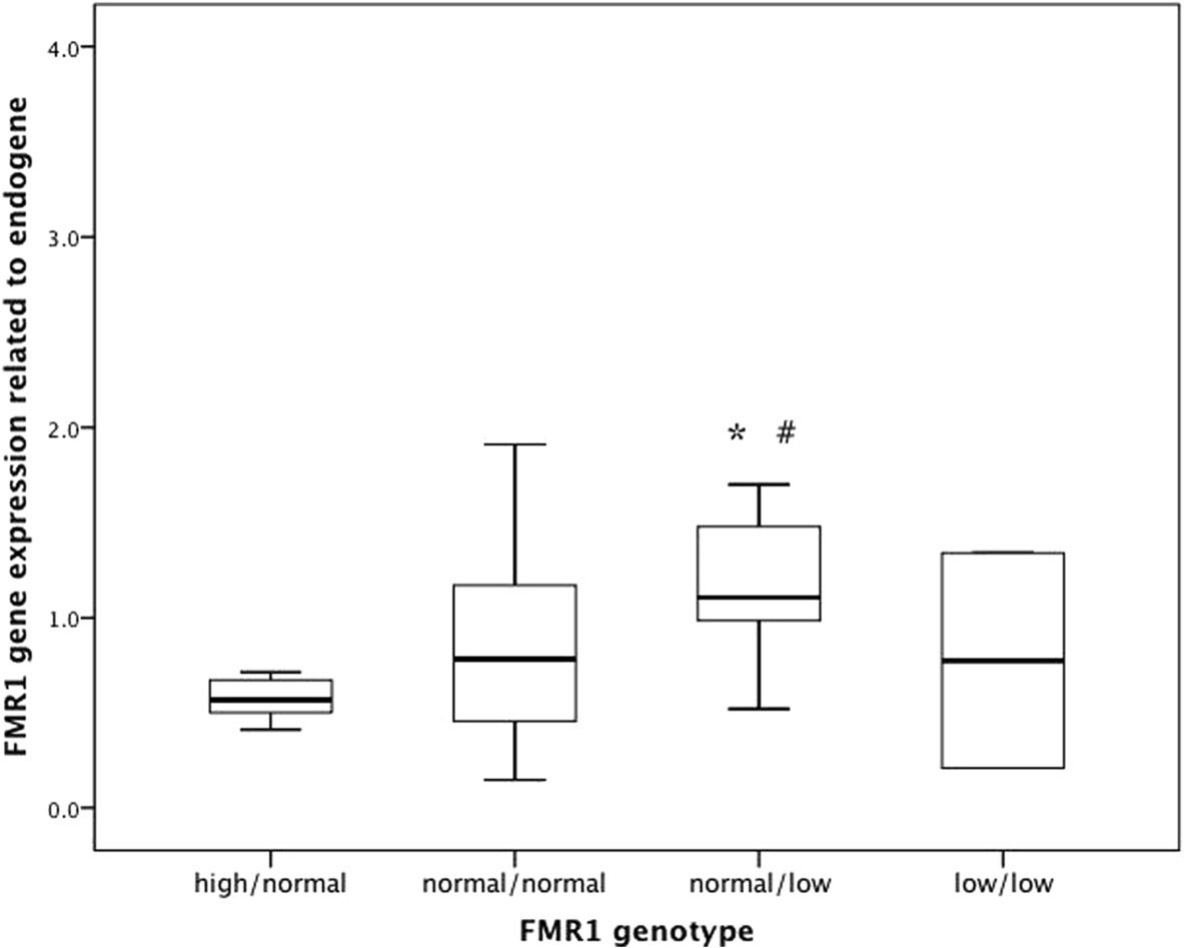
*FMR1* gene expression in poor responder group (POR) depending on *FMR1* genotype. * Statistically different from high/normal group (*p* = 0.008); ^#^ statistically different from normal/normal group (*p* = 0.027)

Further analysis of the different genotypes in POR group demonstrated that the *FMR1* expression in normal/low genotype group is significantly different compared with that in both normal/high or normal/normal genotype groups (*p* = 0.008 and *p* = 0.027, respectively; Fig. 1).

## Discussion

Poor ovarian response is one of the major factors limiting the success rate of infertility treatments. *FMR1* is involved in folliculogenesis and the CGG repeat length in PM range was shown to be associated with the development of POI. An association between high *FMR1* mRNA expression levels in GCs in mid-range *FMR1* PM-carriers and the lowest total number of retrieved oocytes during IVF, compared with the other analyzed groups, was demonstrated already earlier [23] suggesting that via the exact level of *FMR1* expression ovarian response may be judged.

Here, we aimed to analyze the effects of CGG repeat length aberrations outside the PM stage on the *FMR1* expression in human GCs, and its potential correlation with different ovarian response patterns. We detected a significant effect of normal/low CGG repeat length in the POR group on gene expression with significantly elevated *FMR1* mRNA expression levels in this group.

The impact of different *FMR1* CGG repeat lengths below the PM range as a potentially important marker of female fertility and ovarian response remains controversial. Different results obtained by analyzing the non-PM *FMR1* GCC repeat lengths may be partially explained by the difference in study endpoints. While some groups focused on ovarian reserve or response to ovarian stimulation depending on different *FMR1* CGG haplo- and genotypes, others considered the age of menopause or AMH-level as CGG-dependent factors [12–22]. Also, in the pathogenesis of other folliculogenesis disorders the CGG repeat length is supposed to be involved. In PCO patients with normal/low *FMR1* genotype statistically, for example, higher rates of autoimmunity and lower pregnancy rates were detected [33].

In our study the number of PCO patients included was quite low and low alleles were not detected in our PCO group. The low number of PCO in our study can be explained by our clinical procedures, where in vitro maturation (IVM) is the protocol of choice offered to patients with PCO due to an increased risk of developing ovarian hyperstimulation syndrome instead of the classical IVF/ICSI treatment. Patients undergoing IVM treatment were not included in this study. The exclusion of PCO patients from our analysis does not affect the demonstrated results, leading to the same conclusions. Nevertheless, for future studies consideration of *FMR1*-genotype dependent *FMR1*-expression variations in GCs of PCO-patients, especially those with low alleles, may be promising, too.

Furthermore, one of the limitations of our study is, that we did not specifically check for CGG mosaics. Therefore, we cannot exclude existing mosaics in our patients, unless it is described, that only 1% of cases are affected by this [34].

Additionally, we opine that the discrepancies in distribution of ART-treatments between NOR and PCO with POR are of minor importance, since our study evaluated the gene expression in GCs after follicular aspiration prior to fertilization in dependence of ovarian response and *FMR1*-genotype and not the reproductive outcome.

We here evaluated for the first time the putative effects of aberrant non-PM *FMR1* CGG repeat numbers on female fertility based on the individual *FMR1* mRNA gene expression levels in GCs, the *FMR1* target cells in the ovary, that are highly relevant for proper folliculogenesis and oocyte maturation. We thereby aimed to help elucidate the controversially discussed impact of CGG repeat length aberrations on female fertility and ovarian reserve [12–22]. We believe that our findings can contribute to resolve this contentious issue as it combines *FMR1*-gene-expression analysis in GCs with the ovarian response of patients. Our obtained results herein are in line with our previously obtained data, showing different PM-independent *FMR1* expression levels in POI patients [3].

As POR may be considered as a clinical stage putatively leading to the development of POI, we used female GCs and focused on women showing different response to controlled ovarian stimulation, to evaluate the potential association between their *FMR1* mRNA expression and different *FMR1* genotypes, together with different ovarian response patterns directly in female germline.

We demonstrated that patients belonging to the POR group, with a normal/low CGG genotype, show a significantly increased *FMR1* expression levels in GCs, compared with those in the other genotype groups (normal/normal and normal/high). Combined with NOR and PCO such an effect was not detected. This can be due to the limited sample size. So we appreciate further studies to clarify this issue. If such a low allele effect only in POR with larger sample sizes persists, it can be hypothesized, that women with a normal/low CGG repeat length *FMR1* genotype suffer from POR due to their elevated *FMR1* mRNA expression levels that negatively affect the response to controlled ovarian hyperstimulation. Alternatively, poor response of these patients could be the visible endpoint of an already impaired folliculogenesis whose proper process is depending on a sound *FMR1*/FMRP expression regulation. The low-CGG-allele thereby putatively influences this expression regulation. This would be in line with the results of a previous study, that showed in patients carrying heterozygous low CGG repeat length alleles a significantly increased percentage of poor-quality embryo morphology and a lower potential of conceiving after ART [35]. One possible mechanism explaining such a low-allele effect only in POR may be a skewed X Inactivation, as it is described in PM carrier women suffering from FXTAS (Fragile-X-Associated Tremor/Ataxia Syndrome) l [36].

Due to the limited low/low genotype numbers in POR (*n* = 2) and NOR (*n* = 6) we could not detect an effect on the *FMR1* mRNA expression in POR, neither in NOR with two low alleles. So, if this effect is even more pronounced when two low alleles are present, stays speculative. To elucidate this further studies with larger sample sizes are needed, that especially include more patients with homozygous and heterozygous low alleles.

If the CGG repeat length acts as a positive or negative modulator of the *FMR1* translation, as previously hypothesized [11], low CGG repeat numbers may induce gene transcription in POR patients putatively via altered binding capacity of transcriptional factors and/or other regulatory elements such as non coding RNAs (microRNAs, long non coding RNAs), variable CpG methylation and histone modifications. Evaluating these factors in a CGG repeat length depending setting with regards to altered *FMR1* expression levels would therefore be of major interest in future studies.

The elevated *FMR1* mRNA levels of POR with normal/low genotype, similar to the situation in female PM-carriers, may also have a toxic effect and lead to altered FMRP levels. Therefore, in future studies the level of FMRP in dependence of FMR1 genotype, the level of RNA and the ovarian response should be evaluated. In such a study, altered FMRP-level as potential cause of the poor response and negative effects on proper oocyte and embryo development after fertilization might be identified. So further experiments aiming at the reproductive outcome depending on different genotypes, *FMR1/*FMRP expression level and ovarian response are advisable.

In conclusion, analysis of *FMR1* expression in GCs obtained from women with different ovarian reserves can help to obtain a better insight into the *FMR1*/FMRP expression regulatory mechanism and its putative effects on female fertility and folliculogenesis disorders. To the best of our knowledge, we are the first group analyzing the impact of the CGG genotype on the ovarian response and *FMR1* expression directly at the locus of interest in GCs, although larger samples are needed to substantiate the results of this pilot project. Also, functional studies are needed that evaluate involved regulatory elements in order to clarify if and how high mRNA-expression-level of *FMR1* and the CGG repeat length impact follicular maturation and ovarian response.

## Conclusions

Heterozygous low CGG repeat numbers are associated with significantly elevated *FMR1*-expressions profiles in granulosa cells of women with poor ovarian response. If the genotype directly affects female ovarian response or, if this low response is caused by impaired folliculogenesis prior to stimulation due to a low-allele-effect stays speculative. Our results may contribute to a better understanding of *FMR1* expression-regulation in GCs in order to elucidate underlying pathomechanisms of different folliculogenesis disorders and are potentially of value to develop risk-adjusted treatments during IVF/ICSI therapy, in which *FMR1* genotyping provides the better estimate of treatment outcome and allows the optimal adaptation of stimulation protocols in future.

## Abbreviations

AFC: Antral Follicle Count
AMH: Anti-Müllerian Hormone
ART: Assisted Reproductive Techniques
BMI: Body Mass Index
COC: Cumulus Oocyte Complex
E_2_: Estradiol
*FMR1*: Fragile X Mental Retardation I gene
FMRP: Fragile X Mental Retardation I protein
FSH: Follicle Stimulating Hormone
FXPOI: Fragile X-associated POI
FXTAS: Fragile X-associated tremor/ataxia syndrome
GC: Granulosa Cells
GnRH: Gonadotropin-releasing Hormone
ICSI: Intracytoplasmic Sperm Injection
IVF: In vitro fertilization
IVM: In vitro maturation
LH: Luteinizing Hormone
MII: Mature Oocytes
NOR: Normal responders
PCOS: Polycystic Ovarian Syndrome
PM: Pre-mutation
POF/POI: Premature Ovarian Failure/Insufficiency
POR: Poor responders

## References

[1] Barad DH, Weghofer A, Gleicher N (2007). Age-specific levels for basal follicle-stimulating hormone assessment of ovarian function. Obstet Gynecol.

[2] Sullivan AK, Marcus M, Epstein MP, Allen EG, Anido AE, Paquin JJ, Yadav-Shah M, Sherman SL (2005). Association of FMR1 repeat size with ovarian dysfunction. Hum Reprod.

[3] Schuettler J, Peng Z, Zimmer J, Sinn P, von Hagens C, Strowitzki T, Vogt PH (2011). Variable expression of the fragile X mental retardation 1 (FMR1) gene in patients with premature ovarian failure syndrome is not dependent on number of (CGG)n triplets in exon 1. Hum Reprod.

[4] Fu YH, Kuhl DP, Pizzuti A, Pieretti M, Sutcliffe JS, Richards S, Verkerk AJ, Holden JJ, Fenwick RG, Warren ST (1991). Variation of the CGG repeat at the fragile X site results in genetic instability: resolution of the Sherman paradox. Cell.

[5] Devys D, Lutz Y, Rouyer N, Bellocq JP, Mandel JL (1993). The FMR-1 protein is cytoplasmic, most abundant in neurons and appears normal in carriers of a fragile X premutation. Nat Genet.

[6] Kenneson A, Zhang F, Hagedorn CH, Warren ST (2001). Reduced FMRP and increased FMR1 transcription is proportionally associated with CGG repeat number in intermediate-length and premutation carriers. Hum Mol Genet.

[7] Primerano B, Tassone F, Hagerman RJ, Hagerman P, Amaldi F, Bagni C (2002). Reduced FMR1 mRNA translation efficiency in fragile X patients with premutations. RNA.

[8] Hagerman PJ, Hagerman RJ (2004). The fragile-X premutation: a maturing perspective. Am J Hum Genet.

[9] Greco CM, Berman RF, Martin RM, Tassone F, Schwartz PH, Chang A, Trapp BD, Iwahashi C, Brunberg J, Grigsby J (2006). Neuropathology of fragile X-associated tremor/ataxia syndrome (FXTAS). Brain J Neurol.

[10] Greco CM, Hagerman RJ, Tassone F, Chudley AE, Del Bigio MR, Jacquemont S, Leehey M, Hagerman PJ (2002). Neuronal intranuclear inclusions in a new cerebellar tremor/ataxia syndrome among fragile X carriers. Brain J Neurol.

[11] Chen LS, Tassone F, Sahota P, Hagerman PJ (2003). The (CGG)n repeat element within the 5′ untranslated region of the FMR1 message provides both positive and negative cis effects on in vivo translation of a downstream reporter. Hum Mol Genet.

[12] Gleicher N, Weghofer A, Oktay K, Barad D (2009). Relevance of triple CGG repeats in the FMR1 gene to ovarian reserve. Reprod BioMed Online.

[13] Gustin SL, Ding VY, Desai M, Leader B, Baker VL (2015). Evidence of an age-related correlation of ovarian reserve and FMR1 repeat number among women with "normal" CGG repeat status. J Assist Reprod Genet.

[14] Gleicher N, Weghofer A, Barad DH (2010). Ovarian reserve determinations suggest new function of FMR1 (fragile X gene) in regulating ovarian ageing. Reprod BioMed Online.

[15] Pastore LM, Young SL, Baker VM, Karns LB, Williams CD, Silverman LM (2012). Elevated prevalence of 35–44 FMR1 trinucleotide repeats in women with diminished ovarian reserve. Reprod Sci.

[16] Pastore LM, Young SL, Manichaikul A, Baker VL, Wang XQ, Finkelstein JS (2017). Distribution of the *FMR1* gene in females by race-ethnicity: women with diminished ovarian reserve versus women with normal fertility (SWAN study). Fertil Steril.

[17] Gleicher N, Weghofer A, Lee IH, Barad DH (2011). Association of FMR1 genotypes with in vitro fertilization (IVF) outcomes based on ethnicity/race. PLoS One.

[18] Voorhuis M, Onland-Moret NC, Fauser BC, Ploos van Amstel HK, van der Schouw YT, Broekmans FJ (2013). The association of CGG repeats in the FMR1 gene and timing of natural menopause. Hum Reprod.

[19] Banks N, Patounakis G, Devine K, DeCherney AH, Widra E, Levens ED, Whitcomb BW, Hill MJ (2016). Is FMR1 CGG repeat length a predictor of in vitro fertilization stimulation response or outcome?. Fertil Steril.

[20] Maslow BS, Davis S, Engmann L, Nulsen JC, Benadiva CA (2016). Correlation of normal-range FMR1 repeat length or genotypes and reproductive parameters. J Assist Reprod Genet.

[21] Morin SJ, Tiegs AW, Franasiak JM, Juneau CR, Hong KH, Werner MD, Zhan Y, Landis J, Scott RT (2016). FMR1 gene CGG repeat variation within the normal range is not predictive of ovarian response in IVF cycles. Reprod BioMed Online.

[22] Ruth KS, Bennett CE, Schoemaker MJ, Weedon MN, Swerdlow AJ, Murray A (2016). Length of FMR1 repeat alleles within the normal range does not substantially affect the risk of early menopause. Hum Reprod.

[23] Elizur SE, Lebovitz O, Derech-Haim S, Dratviman-Storobinsky O, Feldman B, Dor J, Orvieto R, Cohen Y (2014). Elevated levels of FMR1 mRNA in granulosa cells are associated with low ovarian reserve in FMR1 premutation carriers. PLoS One.

[24] Mailick MR, Hong J, Greenberg J, Smith L, Sherman S (2014). Curvilinear association of CGG repeats and age at menopause in women with FMR1 premutation expansions. Am J Med Genet B Neuropsychiatr Genet.

[25] Ferraretti AP, La Marca A, Fauser BC, Tarlatzis B, Nargund G, Gianaroli L, Definition Ewgo POR (2011). ESHRE consensus on the definition of 'poor response' to ovarian stimulation for in vitro fertilization: the bologna criteria. Hum Reprod.

[26] Rotterdam EA-SPcwg (2004). Revised 2003 consensus on diagnostic criteria and long-term health risks related to polycystic ovary syndrome (PCOS). Hum Reprod.

[27] Fassnacht W, Mempel A, Strowitzki T, Vogt PH (2006). Premature ovarian failure (POF) syndrome: towards the molecular clinical analysis of its genetic complexity. Curr Med Chem.

[28] Stoyanova V, Oostra BA (2004). The CGG repeat and the FMR1 gene. Methods Mol Biol.

[29] Chomczynski P, Sacchi N (1987). Single-step method of RNA isolation by acid guanidinium thiocyanate-phenol-chloroform extraction. Anal Biochem.

[30] Chomczynski P (1993). A reagent for the single-step simultaneous isolation of RNA, DNA and proteins from cell and tissue samples. BioTechniques.

[31] Winer J, Jung CK, Shackel I, Williams PM (1999). Development and validation of real-time quantitative reverse transcriptase-polymerase chain reaction for monitoring gene expression in cardiac myocytes in vitro. Anal Biochem.

[32] van den Berg-Bakker CA, Hagemeijer A, Franken-Postma EM, Smit VT, Kuppen PJ, van Ravenswaay Claasen HH, Cornelisse CJ, Schrier PI (1993). Establishment and characterization of 7 ovarian carcinoma cell lines and one granulosa tumor cell line: growth features and cytogenetics. Int J Cancer.

[33] Gleicher N, Weghofer A, Lee IH, Barad DH (2010). FMR1 genotype with autoimmunity-associated polycystic ovary-like phenotype and decreased pregnancy chance. PLoS One.

[34] Biancalana V, Glaeser D, McQuaid S, Steinbach P (2015). EMQN best practice guidelines for the molecular genetic testing and reporting of fragile X syndrome and other fragile X-associated disorders. Eur J Hum Genet.

[35] Kushnir VA, Yu Y, Barad DH, Weghofer A, Himaya E, Lee HJ, Wu YG, Shohat-Tal A, Lazzaroni-Tealdi E, Gleicher N (2014). Utilizing FMR1 gene mutations as predictors of treatment success in human in vitro fertilization. PLoS One.

[36] Alvarez-Mora MI, Rodriguez-Revenga L, Feliu A, Badenas C, Madrigal I, Milà M (2016). Skewed X inactivation in women carrying the FMR1 Premutation and its relation with fragile-X-associated tremor/ataxia syndrome. Neurodegener Dis.

